# The Interventional Effects of Tubson-2 Decoction on Ovariectomized Rats as Determined by a Combination of Network Pharmacology and Metabolomics

**DOI:** 10.3389/fphar.2020.581991

**Published:** 2020-10-14

**Authors:** Fan Yang, Xin Dong, Feixiang Ma, Feng Xu, Jie Liu, Jingkun Lu, Chunyan Li, Ren Bu, Peifeng Xue

**Affiliations:** ^1^ Department of Pharmacy, Inner Mongolia Medical University, Hohhot, China; ^2^ State Key Laboratory of Natural and Biomimetic Drugs, School of Pharmaceutical Sciences, Peking University, Beijing, China

**Keywords:** network pharmacology, metabolomics, post-menopausalosteoporosis, ovariectomized, Tubson-2

## Abstract

Post-menopausal osteoporosis (PMOP) is associated with estrogen deficiency and worldwide, is becoming increasingly more prevalent in aging women. Various anti-PMOP drugs have been developed to reduce the burden of PMOP; generally, these drugs are efficacious, but with some adverse side effects. Tubson-2 decoction (TBD), a popular traditional Mongolian medicine, has been used to treat PMOP for centuries. However, the precise mechanisms underlying the action of TBD on PMOP have yet to be fully elucidated. Herein, we combined network pharmacology with untargeted metabolomics to identify the key targets and metabolic pathways associated with the interventional effects of TBD on ovariectomized (OVX) rats. Furthermore, we investigated the bone histomorphometry of eight different groups of rats to evaluate the therapeutic effect of TBD. First, we established a TBD-target/PMOP network *via* network pharmacology; this network identified three key protein targets—vitamin D receptor (VDR), cytochrome P450 19A1 (CYP19A1), and 11β-hydroxysteroid dehydrogenase type 1 (HSD11B1). Morphological analysis showed that severe impairment of the bone micro-architecture in OVX rats could be improved by TBD administration. The TBD-treated rats had a significantly lower bone surface-to-tissue volume (BS/TV) and a significantly smaller trabecular separation (Tb·Sp.) (*P*<0.05) than the OVX rats; in contrast, bone volume fraction (BVF), trabecular thickness (Tb·Th.), trabecular number (Tb·N.), and bone mineral density (BMD) were significantly higher in the TBD-treated rats (*P*<0.05). Multivariate and univariate analysis showed that OVX resulted in significant alterations in the concentrations of 105 metabolites and 11 metabolic pathways (P<0.05); in addition, 26 potential biomarkers were identified to investigate the progression of PMOP. Network pharmacology showed that major alterations in vitamin B6 metabolism were associated with the VDR target. Next, we validated the three crucial targets (VDR [P<0.01], HSD11B1 [P<0.01], and CYP19A1 [P<0.05]) by enzyme-linked immunosorbent assays (ELISAs) and demonstrated that the levels of these targets were elevated in the OVX group but reduced in the TBD-treatment group. Collectively, our results suggest that the interventional effects of TBD on OVX rats are likely to be associated with the down regulation of VDR. Our findings enhance our molecular understanding of the interventional effects of TBD on PMOP and will allow us to develop further TBD studies.

## Introduction

Osteoporosis is a systemic bone disease that results in alterations in the microscopic architecture of the bone and a reduction in bone mass (Zou et al., 2020); collectively, these effects result in an impairment of bone quality. Post-menopausal osteoporosis (PMOP) is a global public health issue and has become a significant cause for concern for middle-aged and elderly women (Alexandre and Vico, 2011). Estrogen deficiency is known to be one of the most important mechanisms underlying PMOP and usually occurs 5 to 10 years after menopause (Li et al., 2020). Research studies have also shown that osteoclastic activity exceeds osteoblastic activity after menopause, thus leading to an increase in bone resorption. In turn, these events cause an overall reduction in bone mass reduction and a concomitant increase in skeletal fragility (Gambacciani et al., 2019). Over the last few decades, a considerable body of evidence has accumulated in support of the fact that estrogen prevents bone loss by blocking the production of proinflammatory cytokines by the bone marrow and bone cells. Thus, estrogen replacement therapy remains the gold standard for treating PMOP (Jiang, 2018). However, over the long-term, estrogen replacement therapy is associated with a range of side effects, including pain in the ovaries and endometrium and invasive breast cancer (Komm et al., 2015). Consequently, there is an urgent need to identify novel treatment modalities that are effective but with fewer side effects than existing therapies. Traditional Chinese medicine (TCM), along with other forms of ethnic medicines, has emerged as promising candidates for the treatment of complex disease because these forms of medicine contain multiple components that act on multiple targets. Many researchers have highlighted the fact that many herbs, and prescriptions created from these herbs, can exert and estrogen-like action, including *Eucommia ulmoides* Oliver (EC) (Zhang et al., 2012), *Epimedium brevicornu* Maxim (Liu H. et al., 2018), *Achyranthes bidentata* (Zhang M. et al., 2018), and *Echinops latifolius* Tausch (ELT) (Wang et al., 2020). Considering that these herbs, and their prescriptions, are known to have good anti-osteoporosis effects, it follows that TCM (Li et al., 2018), and other ethnic medicines, may represent a natural drug resource for screening new drugs for the treatment of PMOP.

Tubson-2 (TBD), also known as Erwei Duzhong Decoction, is composed of ELT and EC. This is the basic prescription of Mongolian medicine for treating osteoporosis, recorded in Golden Cabinet of Mongolian Medicine (Zhan, 1977). In a previous study, researchers (Liu, 2016) applied this prescription to 60 patients with PMOP and reported a total effective rate of 90%. In addition, the ameliorating effect of TBD on estrogen receptors, inorganic elements, bone density, bone biomechanics, and bone metabolism, in ovariectomized (OVX) rats have also been investigated in previous research studies (Liu, 2016). Interestingly, ELT was shown to be able to prevent PMOP by activating the estrogen receptor (ER)/protein kinase B (AKT)/extracellumar-signal regulated kinase (ERK) pathway in bone marrow derived mesenchymal stem cells (BMSCs) (Liu Y. et al., 2018), and that the main components of ELT (such as chlorogenic acid and its derivatives) were closely related to oxidative balance in femoral tissue (Han et al., 2017). Moreover, the active compound in EC is known to be a powerful inhibitor of adipogenesis and an enhancer of osteoblastogenesis (Tan et al., 2014). Although the substances that make up ELT and EC have been shown to exert good effects against osteoporosis, the specific mechanisms that underlie the effect of TBD on PMOP have yet to be fully elucidated. However, the complexity of TBD makes its very challenging to investigate its specific mechanism of action. Unlike traditional western medicine, it is not possible to use monomers to perform pharmacodynamic tests with TBD.

Network pharmacology is a new discipline that allows researchers to discover the mechanisms underlying the effects of drug combinations based on multidisciplinary theories such as systems biology and multidirectional pharmacology (Hao and Xiao, 2014). Furthermore, network pharmacology allows us to construct a ‘disease-targets-drug’ network in order to elucidate the mechanisms of action for specific drugs. Metabolomics is considered the latest “omics” science and involves the comprehensive and systematic analysis of small molecular metabolites from tissues, cells, or biological fluids (Johnson et al., 2016). The composition and concentration of metabolites may change with disease progression; this, in turn, will help us to screen potential biomarkers or targeted therapeutic pathways that are closely related to the disease (Lv et al., 2016). Currently, the overall concept of network pharmacology and metabolomics concurs with the ‘multi-component and multi-target’ theory of traditional herbal medicines that is used widely to discover the mechanisms underlying the action of traditional herbal medicines (Zhang et al., 2020).

In the present study, we used a combination of network pharmacology and non-targeted metabolomics to identity and characterize the key targets and metabolic pathways associated with the interventional effects of TBD on PMOP. To verify the efficacy of TBD on PMOP, we created an OVX rat model to mimic the physiological characteristics of menopausal women. First, we established a TBD-target/PMOP network *via* network pharmacology; this network was then used to identify key protein targets. Next, we analyzed relevant metabolites and metabolic pathways by performing ultra-high performance liquid chromatography mass spectrometry (UHPLC-MS) on serum samples acquired from treated and non-treated rats. Finally, we validated the crucial targets of shared pathways identified by network pharmacology and metabolomics to identify the main interventional effects of TBD on OVX rats. We believe that this approach could significantly enhance our understanding of the mechanisms underlying the effect of TBD on PMOP and thus help to promote its clinical application.

## Materials and Methods

### Chemicals and Reagents

*Echinops latifolius* Tausch (ELT) was collected from Wu Chuan (Hohhot, China) and *Eucommia ulmoides* Oliv. (EC) was purchased from Bozhou Pharmaceutical Co. Ltd. (Anhui, China). Chloral hydrate, combined estrogen tablets, Gushukang granules, and saline, were purchased from Ze Sheng Biotechnology Company (Hohhot, China). Methanol and formic acid (MS grade) were acquired from Fisher Scientific Corporation (Loughborough, UK). Ultra-high purity water (18.2 MΩ) was obtained from an ALH-600-U purification system (Chongqing, China). Enzyme-linked immunosorbent assay (ELISA) kits for the vitamin D receptor (VDR), cytochrome P450 19A1 (CYP19A1), 11β-hydroxysteroid dehydrogenase type 1 (HSD11B1) were obtained from Hua Lian Biotechnology Institute (Wuhan, China).

### Instrumentation

The research described herein utilized the following equipment: a high resolution *in-vivo* X-ray microtomograph (SkyScan 1176, Bruker, Germany), a digital microscope (MOTICBA210, Motic, China), an ultrapure water system (ALH-600-U, China), a Thermo Scientific Q Exactive Quadrupole-Orbitrap Mass Spectrometer System (Thermo Fisher Scientific Inc, Grand Island, NY, USA), a rotary evaporator (EYELA n-1100, Shanghai Alang Instruments Co., China), and a microplate reader (Beijing Prolong New Technology Co., China).

### Plant Materials and Preparation of Tubson-2 Decoction

Herbal materials of ELT were collected from Wu Chuan (Hohhot, China, GPS co-ordinates of the plant collection site is 41.1206962700, 111.4084477500) in early autumn 2019 and identified by Professor PX (Department of Natural Medicinal Chemistry, Inner Mongolia Medical University) with the voucher specimen (#20190715) collected in the herbarium of Inner Mongolia Medical University. EC herbal materials were purchased from Bozhou Pharmaceutical Co. Ltd. (Anhui, China) and morphologically authenticated by PX. The voucher specimens were also deposited in the herbarium of Inner Mongolia Medical University (#20190822).

The final preparation of TBD was carried out as described in our previous paper. In brief, two herbal materials were ground into powder and screened over 50 mm sieve before use. Equal amounts of ELT and EC powders were mixed and then were soaked with ultrapure water at a material-liquid ratio of 1:10 (g/ml) for half an hour before being hot reflux extraction. Subsequently, these powders were refluxed at 100°C twice (for 30 min each time), and then filtered through 180 mm sieve. Following filtration, the filtrate was collected, concentrated, and freeze-dried to obtain a lyophilized TBD powder. Powders were stored in a refrigerator at 4°C and dissolved in an appropriate amount of distilled water prior to use. The chemical proﬁle of TBD was achieved by UPLC-MS/MS (Supporting Information **Figure S1**). The content of sixteen active ingredients in TBD sample was list as **Supporting Information Table S1**.

### Establishment of an OVX Rat Model

**Figure 1:**
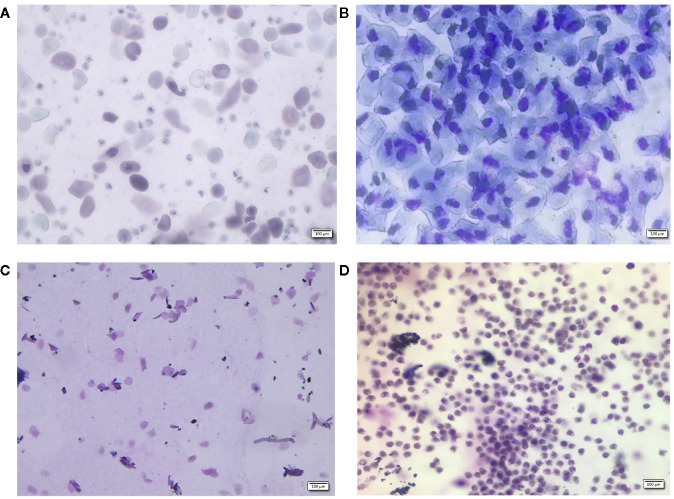
Vaginal morphology in sham-operated **(A–C)** rats and ovariectomized (OVX) rats **(D)**. Methylene blue for 10 min, ×100. Sham group rats had an estrous cycle in general, **(A)**: nucleated epithelial cells, keratinized epithelial cells, and leukocytes appear evenly to show a state of the estrous, **(B)**: a large number of nucleated epithelial cells show the a state of proestrus period, **(C)**: a lot of keratinized epithelial cells show the a state of estrus period, **(D)**: a large number of non-nucleated leukocytes show the a state of diestrus period.

Twelve-week-old female Wistar rats, 250–270 g in weight, were purchased from the Laboratory Animal Institute of Inner Mongolia Medical University (Hohhot, China). All animal procedures were approved by the Animal Ethics Committee of Inner Mongolia Medical University (Reference: SCXK2015-0001). Rats were kept in a barrier system with controlled temperature (25–28°C) and humidity (50%–60%) and a light-dark cycle of 12 h per day. After 7-day of acclimatization, 48 rats were equally randomized to 8 groups (6 rats per group), including a blank group, a sham group, an OVX group, a positive control drug group (Gushukang-treated group and an estrogen-treated group), a TBD high dose group, a TBD middle dose group, and a TBD low dose group. All rats except those in the blank group were injected with 10% chloral hydrate solution (0.5 ml/100 g body weight) prior to surgery. Bilateral laparotomy was conducted in the sham group (n=6) while bilateral ovariectomy was carried out in the model group (n=36, six rats were regarded as the OVX group, while the remaining rats were allocated to drug-treatment groups). Seven days after surgery, all rats were subjected to keratinization experiments involving the vaginal epithelium; vaginal smears were then performed once a day for 5 consecutive days. The morphology and staining of the cells contained within vaginal smears were observed by optical microscopy at a magnification of 100×. Results showed that the estrus cycle appeared alternately in the blank group (**Figures 1A–C**), while an estrus interval appeared in the model group (**Figure 1D**) five consecutive days of vaginal smear to tips for successful modeling.

The low dose group was given 237.5 mg·kg^-1^·d^-1^ of lyophilized TBD (equivalent to 1.05 g·kg^-1^·d^-1^ of raw medicine), the middle dose group was given 475 mg·kg^-1^·d^-1^ (equivalent to 2.1 g·kg^-1^·d^-1^ of raw medicine), and the high dose was given 950 mg·Kg^-1^·d^-1^ (equivalent to 4.2 g·kg^-1^·d^-1^ of raw medicine). The Gushukang-treated group was given Gushukang granules (105.1 mg·kg^-1^·d^-1^) while estrogen-treated group was given combined estrogen tablets (0.065 mg·kg^-1^·d^-1^). During the same period, the blank group, sham group, and OVX group, were given the same volume of 0.9% saline daily. After 8 weeks of gavage treatment, we anesthetized all animals with 10% chloral hydrate solution and acquired cardiac blood samples. Serum was separated by centrifugation at 3,500 rpm for 10 min at 4°C and frozen at -80°C to await metabolomics analysis. We also removed the left tibia from each animal and fixed these samples in formalin to await analysis.

### Analysis of Bone Mineral Density and Trabecular Micro-Architecture

X-ray microtomography was used to analyze bone mineral density and trabecular micro-architecture. All tibias were fixed with 70% ethanol and subjected to X-ray microtomography with an isotropic voxel size of 10 μm. Tomographic images were acquired at an integration time of 250 ms with 500 projections over the full 360° rotation. Three dimensional reconstructions were generated with the following parameters: smoothing was set as 3; ring artifacts reduction was set to 5; and beam hardening correction was set to 30%. Bone volume fraction (BVF), bone surface to tissue volume (BS/TV), trabecular thickness (Tb·Th.), trabecular number (Tb·N.), trabecular separation (Tb·Sp.), and bone mineral density (BMD), were all determined by analyzing a specific region of interest (ROI) that was chosen by setting the same coordinates in the tibial growth plate for each sample.

### Network Pharmacology Analysis

#### Compound Data Acquisition

Details relating to the compounds identified in ELT and EC were obtained by searching the Traditional Chinese Medicine Systems Pharmacology Database and Analysis Platform (TCMSP; http://tcmspw.com/tcmsp.php) (Ru et al., 2014), the Encyclopedia of Traditional Chinese Medicine (ETCM; http://www.nrc.ac.cn:9090/ETCM/) (Xu et al., 2019), and existing literature (Wang et al., 2020). We also added some additional compounds, as determined by the fingerprint we created for TBD in a previous study (Guo et al., 2020).

#### Target Fishing

We used a systemic approach that was based on information integration and text-mining to identify the targets of TBD. Targets were retrieved from Swiss Target Prediction (http://www.swisstargetprediction.ch) (Gfeller et al., 2014) and Superpred Webserver (http://prediction.charite.de/) (Nickel et al., 2014) software. The SMILE structure of each component was then introduced into Swiss Target Prediction with a probability threshold of 0.6–1. All known and predicted targets were then selected from the Superpred Webserver. Next, the targets that were related to PMOP were acquired from Drugbank (http://www.drugbank.ca/atc) (Wishart et al., 2018). We then constructed a compound-target network and then removed the components of TBD that were not connected with PMOP targets.

#### Construction of a Protein-Protein Interactions (PPI) Network Related to PMOP

The String database (https://string-db.org/Version 10.5) is a database that is commonly used to analyze known and predicted protein-protein interactions. The common targets of PMOP, along with the active ingredients of TBD, were introduced into the database for protein-protein interaction (PPI) analysis and the species set to “human.” We then imported node 1, node 2, and comprehensive scoring information, into Cytoscape 3.0 software to create a PPI network map.

#### Determination of Key Targets

By filtering common targets, it was possible to identify the specific pathways of the targets *via* the DAVID database (https://david.ncifcrf.gov/). Next, using the potential targets of TBD for PMOP, we performed enrichment analysis of biological processes using the CLUE GO plugin in Cytoscape software. We then considered this data along with the rankings for biological functions and pathways and identified important pathways. Next, we ranked the genes associated with the identified pathways in terms of frequency and integrated the target data into the PPI network diagram. Finally, we defined the key targets of TBD in PMOP that would be subsequently assayed quantitatively by enzyme-linked immunosorbent assays (ELISAs).

### Metabolomics Analysis

#### Preparation of Serum Samples and Quality Control (QC) Samples

Serum samples were thawed at 4°C prior to analysis. Then, 200 μl of each serum sample was mixed with 600 μl of methanol and vortexed for 3 min. Subsequently, the samples were centrifuged at 15,000 rpm for 10 min at 4°C. Supernatants were then transferred to new microcentrifuge tubes and dried in a vacuum dryer. Finally, the samples were dissolved in 200 μl of methanol for analysis.

Quality control (QC) samples were created by pooling serum samples from each analysis sequence. QC samples were analyzed after every 10 samples. This practice ensured that our analysis was accurate and consistent.

#### UHPLC-MS Conditions

UHPLC-MS analysis was performed on a Thermo Q-Exactive-MS (Thermo, USA). Chromatographic separations were performed on a Waters ACQUITY UPLC HSS T3 column (2.1×50 mm, 1.8 μm) at 35°C, and the flow rate was set to 0.4 ml/min. The mobile phase was formed by MS-grade methanol (A) and water containing 0.1% formic acid (B). The gradient program was set to the following parameters: 0–1.7 min, 5%–10% A; 1.7–3.0 min, 15%–17% A; 3.0–3.3 min,17% A; 3.3–8.0 min, 17%–25% A; 8.0–9.7 min, 25%–30% A; 9.7–10.6 min, 30%–35% A; 10.6–14.1 min, 35%–55% A, 14.1–14.6 min, 55% A; 14.6–15.1 min, 55%–100% A; 15.1–17.0 min, 100% A; 17.0–18.1 min, 100%–5% A; 18.1–20 min, 5% A. For each sample, we injected 5 µl for analysis.

Q-Exactive-MS analysis was performed in full scan/dd ms^2^ mode. Spray voltage was set to 4 kV for positive ion mode and 3.2 kV for negative ion mode. The sheath gas flow rate was 40 L/min for positive ion mode and 35 L/min for negative ion mode. The auxiliary gas flow was 2 L/min for both of the ion mode sat 350°C. The capillary temperature was 300 V for both two ion modes. Data were collected in centroid mode and the mass range was 100–1,100 m/z.

#### Data Processing and Statistical Analysis

Original data files were independently processed by Compound Discoverer (CD) ™ 2.0 software for peak alignment, peak filter, peak extraction, and automatic integration. The minimum peak intensity for component extraction was 2×10^6^ and the mass tolerance was 10 ppm. The retention time window was 1 min and the signal-to-noise ratio was 3. CD software was then used for spectral comparison, grouping, compound detection, and metabolite identification. We were then able to create multi-dimensional peak tables containing prediction formulas, accurate mass data, retention times, peak areas, and mzCloud results. The multi-dimensional peak tables acquired in positive and negative ion mode were processed separately and transferred into a Microsoft Excel file which was subsequently imported into SIMCA-P (version 14.1, Umetrics, Sweden) Performed principal component analysis (PCA) was then performed to identify the overall metabolic profile for each group of samples. Supervised partial least squares discriminant analysis (PLS-DA) was then applied to compare distinguishing markers between the OVX group and the sham group. The fitness and predictive ability of the model were then verified according to R2 and Q2 values in cross-validation and permutation tests, respectively. We then identified potential biomarkers as metabolites with *P* < 0.05 and variable importance plot (VIP) > 1.

Potential biomarkers were then matched with structural data acquired from the Human Metabolome Database (HMDB) (http://www.hmdb.ca/) and mzCloud (https://www.mzcloud.org/). Pathway enrichment analysis was then performed for the identified biomarkers using MetaboAnalyst (http://www.metaboanalyst.ca/).

### Analysis of Biochemical Indicators

Blood samples were collected from all rats at sacrifice, as described earlier. The serum levels of VDR, HSD11B1, and CYP19A1, were then determined byenzyme-linked immunosorbent assays (ELISAs) in accordance with the manufacturer’s instructions.

### Statistical Analysis

Data relating to BVF, BS/TV, Tb·N., Tb·Sp., Tb·Th., BMD, CYP19A1,VDR, and HSD11B1, are described as the mean ± standard deviation (SD). The student’s unpaired t test was performed with SPSS 19.0 software and a P value ≤ 0.05 was regarded as statistically significant.

## Results

### TBD Improved Ovariectomy-Induced Bone Loss and Deleterious Changes in the Trabecular Micro-Architecture

**Figure 2:**
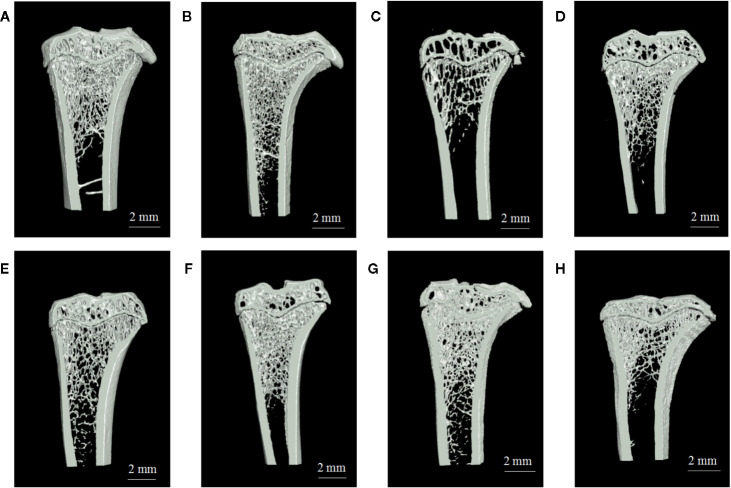
The region of interest (ROI) image and bone parameters analysis in tibia in different rats: **(A)** sham group, **(B)** blank group, **(C)** ovariectomized (OVX) group, **(D)** low dose treated group, **(E)** medium dose treated group, **(F)** high dose treated group, **(G)** gushukang treated group, and **(H)** estrogen treated group.

**Figure 3:**
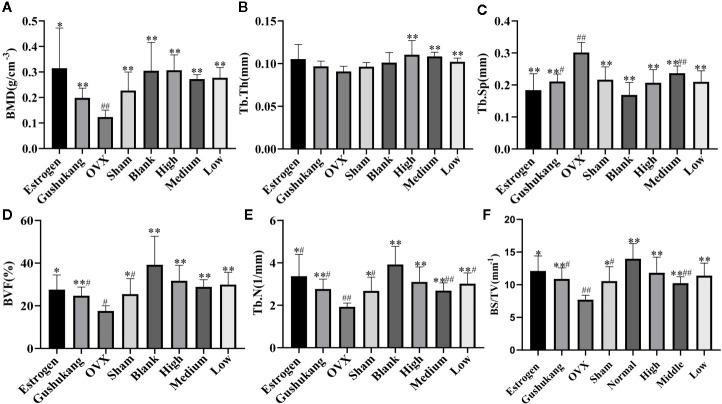
**(A)** Comparison of bone mineral density (BMD) in eight groups; **(B)** Comparison of Tb•Th in eight groups; **(C)** Comparison of Tb•Sp in eight groups; **(D)** Comparison of bone volume fraction (BVF) in eight groups; **(E)** Comparison of Tb•N in eight groups; **(F)** Comparison of BS/TV in eight groups. Values were expressed as the mean ± SD; n = 6. ^#^p < 0.05, ^##^p < 0.01 compared with blank group. *p < 0.05, **p < 0.01 compared with OVX group.

Analysis of vaginal smears from our experimental rats proved that the PMOP model had been successfully established by ovariectomy. Three dimensional microtomographic analyses allowed us to gather additional architectural, cortical, and trabecular, information from the tibias of experimental rats. We found that the OVX group showed significant changes in both BMD and trabecular micro-architecture. As shown in **Figure 2C**, the bone microstructure of the tibia in the OVX group exhibited severe damage. Moreover, the trabecular region in the OVX rats appeared to be small, thin and sparse. Analysis also revealed that there was a significant reduction in Tb·N. (*P*=0.003), BVF (*P*=0.011), BMD (*P*=0.008), and BS/TV (*P*=0.001), along with a significant increase in Tb·Sp. (*P*=0.000) in the OVX group (**Figures 3A–F**).

One striking observation arising from our analysis of bone mineral density and trabecular micro-architecture was the existence of a severe reversion in bone loss in the treatment groups (**Figures 2A–H**). All treatment groups showed a remarkable improvement in trabecular micro-architecture, with a slightly dilated medullary cavity and a slightly disordered trabecular arrangement (**Figures 2D–H**). The trabecular morphological parameters were turned over in these drug-treated groups as shown in **Figures 3A–F**. Compared with the OVX group, the drug-treatment groups showed significant elevations of Tb·N. (*P*<0.05), BVF (*P*<0.05), BMD (*P*<0.05), and BS/TV (*P*<0.05), and a reduction of Tb·Sp. (*P*<0.05). There was only a marginal and non-significant difference in the estimated trabecular morphological parameters when compared between different drug-treatment groups. This data demonstrated that TBD has similar effects in OVX rats as the positive drug. However, the effect of TBD on these indicators did not show a dose-dependent effect.

### VDR, HSD11B1, and CYP19A1 Were Crucial Targets in the Interventional Effects of TBD on OVX Rats

Following the screening of compound data, we identified 195 compounds in TBD, of which 28 were unique to ELT, 163 were unique to EC, and 4 were common to both herbs. Because the oral bioavailability (OB) and drug-likeness (DL) values for most compounds in EC and ELT were low or unknown, we were not able to apply these values for compound screening. We therefore considered all compounds with known targets as the active ingredients of EC and ELT. In total, 62 compounds had known targets; these were considered as the active ingredients of TBD.

**Figure 4:**
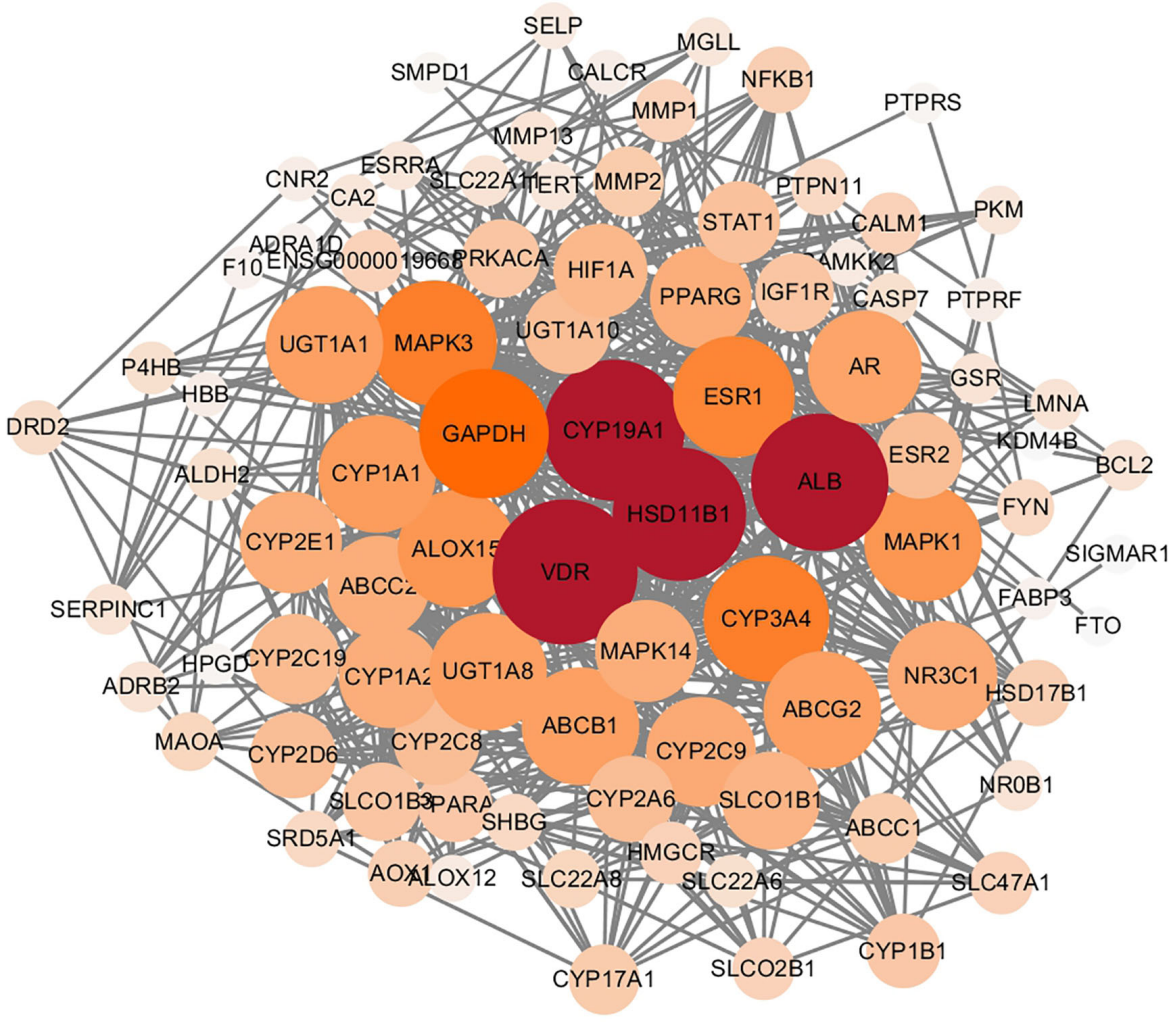
Protein interaction network of Tubson-2 decoction (TBD) against post-menopausal osteoporosis (PMOP). The nodes in the graph represent targets, while the edges represent the association between proteins. And the node size represented degree value. The larger the node, the larger the degree value.

Following the removal of duplicates, we identified a total of 2,251 targets for TBD. In total, we identified 1,412 targets for the active ingredients of EC, 509 targets for the active ingredients of ELT, and 330 targets for the targets shared by EC and ELT. All compound targets were retrieved from Swiss Target Prediction software and the Superpred Webserver. We also identified 581 gene targets that were associated with PMOP *via* the use of Drugbank. After comparing the targets that were associated with both TBD and PMOP, we identified 89 potential targets for TBD against PMOP. These potential targets were entered into the String database (set to human and a high confidence level of 0.7). Node1 and node 2 were then imported into Cytoscape software to allow us to create an interaction network (**Figure 4**). The nodes shown in **Figure 4** graph represent the targets while the edges represent associations between different proteins. The color and size of the nodes reflect the degree value for each protein target: the larger and darker the node, the greater the degree value. As shown in **Figure 4**, the target interaction network featured 89 nodes and 626 edges. According to degree values, the key targets for TBD against PMOP were CYP19A1, HSD11B1, VDR, and albumin (ALB).

GLUE GO analysis identified the top biological features of our interaction network as hormone metabolic process, steroid metabolic process, and oxidoreductase process. We then calculated the gene frequencies for the key target genes in these three biological pathways. Analysis showed that VDR, CYP19A1 and beta-2 adrenergic receptor (ADRB2) appeared eight times; tyrosine-protein phosphatase non-receptor type (PTPN), estradiol 17-beta-dehydrogenase 1 (HSD17B1), and ethylene-responsive transcription factor ESR1 (ESR1) appeared seven times; and HSD11B1 appeared six times. By combining these results with the PPI data, we found that CYP19A1, HSD11B1, and VDR were the most important potential targets for TBD when used to treat PMOP.

### Metabolomics Analysis Showed That 26 Potential Biomarkers Were Involved in OVX-Induced Osteoporosis and Regulated by TBD

**Figure 5:**
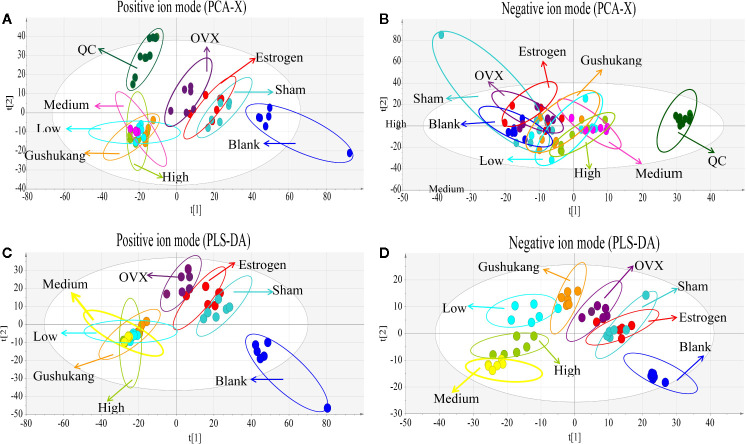
Multivariate analysis based on the UHPLC- Q-Exactive-MS profiling data for samples in the blank, sham, ovariectomized (OVX), low dose treated group, medium dose treated group, high dose treated group, estrogen treated group, and gushukang treated group in positive and negative ion mode (n = 6). **(A)** Principal component analysis (PCA) score plot in positive ion mode; **(B)** PCA score plot in negative ion mode; **(C)** Partial least squares discriminant analysis (PLS-DA) score plot in positive ion mode; **(D)** PLS-DA score plot in negative ion mode.

As shown in the PCA score plot (**Figures 5A, B**), the analysis of QC samples showed good reproducibility and reliability. PCA showed that the dataset derived from the OVX group was separated from the data derived from the other groups. The widespread distribution of data following PCA was likely to be caused by individual differences in rats and/or the timing of sampling. To exclude the effect of potential confounding variables that were not related to the group differences, and to evaluate the statistical significance of these effects, we applied PLS-DA analysis (**Figures 5C, D**). The results of this analysis were highly encouraging as the discrimination model could readily differentiate between the eight groups. Although the distribution of pre-cachexia overlapped with the OVX and estrogen groups, these models showed appropriate goodness-of-fit values with high cross-validation predictability, with cumulative R2Y values of 1.000 and cumulative Q2 values of 0.977 in the positive mode, and R2Y value of 0.959 and a cumulative Q2 value of 0.541 in the negative mode (**Table 1**), indicating reliable differentiation between the groups.

**Table 1:** Summary of partial least squares discriminant analysis (PLS-DA) model parameters for evaluating model quality by 200 permutation tests of corresponding validation plots.

	Ion mode	*A*	R2X	R2Y	Q2(cum)	R2 intercepts	Q2 intercepts
OVX vs Sham	Positive mode	2	0.413	0.998	0.939	0.921	-0.237
	Negative mode	2	0.361	0.956	0.517	0.88	-0.0204
OVX vs Mediuim-treated	Positive mode	2	0.620	0.999	0.978	0.802	-0.0681
	Negative mode	2	0.420	0.997	0.935	0.895	-0.0716
OVX VS Estrogen-treated	Positive mode	2	0.552	1.000	0.977	0.868	-0.0496
	Negative mode	2	0.313	0.959	0.541	0.914	-0.0144

A was number of components.

**Figure 6:**
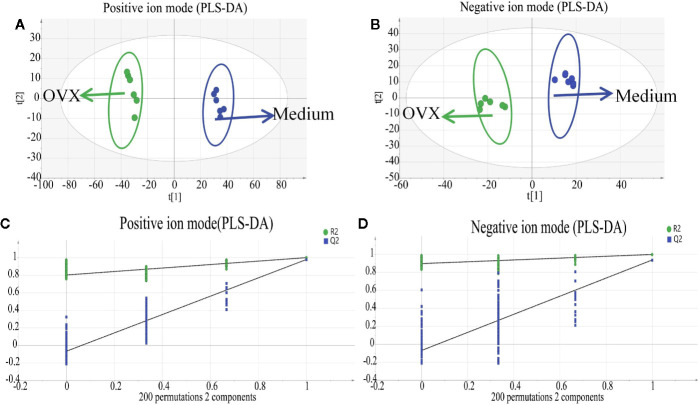
Multivariate analysis based on the UHPLC- Q-Exactive-MS profiling data for samples in the Tubson-2 decoction (TBD) medium-treated and ovariectomized (OVX) group in positive and negative ion mode (n = 6). **(A)** Partial least squares discriminant analysis (PLS-DA) score plot in positive ion mode; **(B)** PLS-DA score plot in negative ion mode. **(C)** permutation test of the TBD medium-treated and OVX group in positive ion mode; **(D)** permutation test of the TBD medium-treated and OVX group in negative ion mode.

In order to identify distinct biomarkers that may be associated with TBD treat OVX-induced osteoporosis among a dataset featuring thousands of variables, we conducted pairwise comparisons between the OVX and TBD medium-treated groups (**Figures 6A, B**). Validation analysis was satisfactory with regards to group classification, with a cumulative R^2^Y value of 0.999 and a cumulative Q^2^ value of 0.978 in the positive mode, and a cumulative R^2^Y value of 0.997 and a cumulative Q^2^ value of 0.935 in the negative mode (**Table 1**). Permutation tests for the TBD medium-treated groups and OVX group are shown in **Figures 6C, D**. After filtering with VIP values, and results arising from the Student’s t test, we identified 105 metabolites as potential biomarkers (82 metabolites from the positive ion mode and 23 metabolites from the negative ion mode). Next, we used HMBD and the mzCloud online database to identify these potential biomarkers. By considering the metabolites that overlapped each pairwise comparison, we identified a total of 26 putative biomarkers (**Table 2**). The potential differential metabolites were identified by standard, ferulic acid, 350 its parent ion is [M-H]-: 193.005063, the main product ion fragments are (m/z): 134.03546, 351 149.06041, 178.00696, and the retention time is 11.45 min. This is consistent with our results and 352 further confirms the reliability of the ferulic acid test results.

**Table 2:** Identification of potential differential metabolites in rats serum in positive and negative mode.

NO.	Metabolite	Formula	Adduct	Accurate Mass (m/z)		Error*(ppm)	RT(min)	VIP	(OVX)/(Sham)	(Medium treated)/(OVX)
				measured	predicted					
1	Monobutyl phthalate	C_12_H_14_O_4_	[M-H]-	223.0964	222.0888	-4.51	1.77	1.80	↑	↓
2	4-Methylhippuric acid	C_10_H_11_NO_3_	[M-H]-	192.0666	193.0724	-5.24	6.60	1.20	↑	↓
3	N-Isovalerylglycine	C_7_H_13_NO_3_	[M-H]-	158.0822	159.0878	-6.36	4.79	1.48	↑	↓
4	Phenobarbital	C_12_H_12_N_2_O_3_	[M-H]-	231.0775	232.0836	-4.35	8.02	1.46	↑	↓
5	Indole-3-acrylic acid	C_11_H_9_NO_2_	[M+H]-	188.0706	187.0631	5.35	13.48	1.45	↓	↑
6	13S-hydroxyoctadecadienoic acid	C_18_H_32_O_3_	[M-H]-	295.2279	296.2346	-3.41	18.10	1.44	↑	↓
7	N-Formylmethionine	C_6_H_11_NO_3_S	[M-H]-	176.0387	177.0445	-5.71	1.72	1.42	↑	↓
8	N-Isobutyrylglycine	C_6_H_11_NO_3_	[M-H]-	144.0666	145.0722	-6.98	1.93	1.38	↑	↓
9	3-Hydroxybutyric acid	C_4_H_8_O_3_	[M-H]-	103.0400	104.0456	-9.75	0.82	1.36	↑	↓
10	12-Hydroxydodecanoic acid	C_12_H_24_O_3_	[M-H]-	215.1653	216.1713	-4.68	17.8	1.25	↑	↓
11	Ferulic acid	C_10_H_10_O_4_	[M-H]-	193.0506	194.0558	-5.21	11.46	1.22	↑	↓
12	Testosterone glucuronide	C_25_H_36_O_8_	[M+H]^+^	465.2483	464.2409	2.17	17.64	1.52	↑	↓
13	1,2-Dipalmitoylphosphatidylglycerol	C_38_H_75_O_10_P	[M+H]^+^	723.5171	744.4980	-2.90	20.67	1.52	↑	↓
14	Kynurenine	C_10_H_12_N_2_O_3_	[M+H]+	209.0921	208.0844	4.82	2.84	1.41	↑	↓
15	Spectinomycin	C_14_H_24_N_2_O_7_	[M+H]+	333.1656	332.1624	3.01	16.49	1.37	↑	↓
16	DL-Carnitine	C_7_H_15_NO_3_	[M+H]^+^	162.1125	161.1047	6.22	1.13	1.35	↓	↑
17	Phenylalanine	C_9_H_11_NO_2_	[M+H]^+^	166.0863	165.0786	6.07	3.24	1.34	↑	↓
18	Phenylacetylglycine	C_10_H_11_NO_3_	[M+H]^+^	194.0812	193.0735	5.19	6.20	1.34	↑	↓
19	Hippuric acid	C_9_H_9_NO_3_	[M+H]+	180.0655	179.0582	5.59	5.78	1.32	↑	↓
20	6-Methylquinoline	C_10_H_9_N	[M+H]^+^	142.0662	143.0733	7.09	5.12	1.29	↓	↑
21	Skatole	C_9_H_9_N	[M+H]+	132.0808	131.0735	7.63	5.12	1.28	↑	↓
22	2-Amino-1,3,4-octadecanetriol	C_18_H_39_NO_3_	[M+H]+	318.3003	317.2921	3.17	18.88	1.10	↓	↑
23	Pyridoxal	C_8_H_9_NO_3_	[M+H]+	168.0655	167.0581	5.99	1.78	1.09	↓	↑
24	Pyridoxamine	C_8_H_12_N_2_O_2_	[M+H]+	169.0972	168.0872	5.97	1.45	1.07	↑	↓
25	3-Hydroxyanthranilic acid	C_7_H_7_NO_3_	[M+H]^+^	154.0499	153.0427	6.54	1.13	1.06	↑	↓
26	Creatine	C_4_H_9_N_3_O_2_	[M+H]+	132.0768	131.0694	7.63	1.77	1.05	↑	↓

*The error between predicted accurate mass and measured accurate mass; RT, retention time; (↓):down-regulated, (↑):up-regulated.

### Metabolomic Analysis Identified Vitamin B6 Metabolism as the Key Pathway in OVX-Induced Osteoporosis and Could Be Regulated by Tubson-2

**Figure 7:**
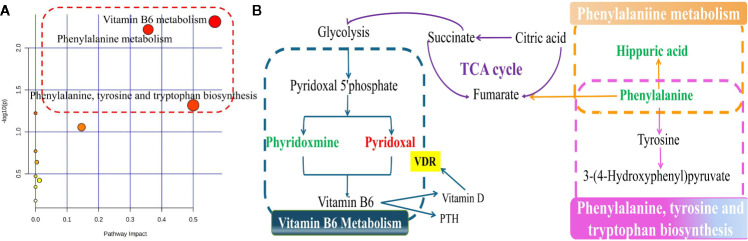
The signaling pathway analysis based on the potential biomarkers **(A)** Summary of ingenuity pathway analysis with MetaboAnalyst. The size and color of each circle were based on pathway impact value and p-value, respectively; **(B)** Construction of the main metabolic pathways related to differential metabolites. (down-regulated in green, up-regulated in red.)

Next, we pooled the metabolites that showed alterations in rat serum and identified a total of 26 putative metabolites for analysis. The fold-changes of these metabolites in the OVX, sham, and medium-dose-treated groups are shown in **Table 2**. Pathway analysis, carried out using MetaboAnalyst (http://www.metaboanalyst.ca/), revealed the detailed impacts of OVX-related alterations in metabolic networks (**Figure 7A**). To investigate the most influential metabolic pathway, we set the following threshold values: -log (*P*) values >1.5 and pathway impact >0.1. Three metabolic pathways were defined as being disturbed in the serum profiles of rats with TBD treated OVX-induced osteoporosis: vitamin B6 metabolism, phenylalanine metabolism, phenylalanine, and tyrosine and tryptophan biosynthesis. Based on the biomarkers that were potentially responsible for TBD treatment, we then used the Kyoto Gene and Genome Encyclopedia database (KEGG (http://www.genome.ad.jp/kegg/), and the human metabolism database (http://www.hmdb.ca/), to create a metabolic network for metabolic pathways that were altered by TBD treated OVX (**Figure 7B**).

### Target Validation Was Performed by ELISA Assay

**Figure 8:**
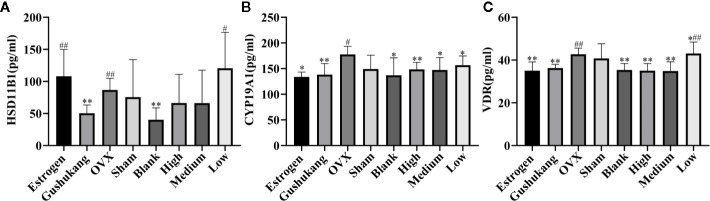
Three targets levels of serum in eight groups: **(A)** HSD11B1; **(B)** CYP19A1; **(C)** VDR. Values were expressed as the mean ± SD; n = 6. ^#^p < 0.05, ^##^p < 0.01 compared with blank group. *p < 0.05, **p < 0.01 compared with OVX group.

We used network pharmacology and metabolomics to identify the crucial targets for TBD in the treatment of PMOP; the most important targets were VDR, HSD11B1, and CYP19A1. We then used ELISA kits to validate the levels of these crucial targets in samples of rat sera. As shown in **Figure 8**, the concentrations of VDR, HSD11B1, and CYP19A1 were elevated in the OVX group, thus presenting the inhibition of these cytokines. The serum levels of VDR and CYP19A1 were reduced following the administration of TBD, suggesting that TBD is able to treat OVX-induced osteoporosis by ameliorating the levels of VDR and CYP19A1.

## Discussion

Previous research (Li et al., 2016) has demonstrated that PMOP is related to estrogen withdrawal and represents the most common metabolic bone disease in females. The use of bilateral ovariectomies in female rats is now well established as the method of choice with which to create an experimental model of osteoporosis (Thompson et al., 1995). Ovariectomy simulates menopause and osteopenia in experimental rats. Although it is well known that ovariectomy induces a menopausal status and ‘osteoporotic’ bone changes (Saleh et al., 2020), we analyzed vaginal smears and performed X-ray microtomography on each of our ovariectomized rats in order to confirm that the OVX-induced model of osteoporosis had been successfully created (Yousefzadeh et al., 2020). We selected Gushukang granules, as a traditional Chinese medicine (TCM) (Li et al., 2019), for osteoporosis, and estrogen tablets, a common supplement for PMOP patients, as positive control drugs with which to evaluate the efficacy of TBD on PMOP. As illustrated in previous, there was only a marginal and non-significant difference between different drug-treatment groups with respect to several trabecular morphological parameters. This implies that TBD exerts the same effect on OVX rats as the positive control drugs. We also investigated the relative effects of different TBD doses on OVX rats; doses were selected according to the guiding principles laid down by non-clinical pharmacokinetic drug research. The “low-dose” of TBD was the clinical equivalent dose, the “medium-dose” of TBD was double the clinical equivalent dose, and the “high-dose” of TBD was four times the clinical equivalent dose. However, we observed no dose-dependent relationship with regards to the relative effects of TBD on trabecular morphological parameters. It is likely that this observation was related to individual differences among the rats.

Metabolomics analysis revealed that 26 putative metabolites and 11 metabolic pathways were associated with the progression of TBD-treated-PMOP, including vitamin B6 (Hellmann and Mooney, 2010) metabolism, phenylalanine metabolism, phenylalanine, and the biosynthesis of both tyrosine and tryptophan. The metabolites involved in these metabolic pathways were shown to interact with each other to form a complex network. The derivatives of pyridoxal and pyridoxamine are able to form vitamin B6; consequently, these are key metabolites in the vitamin B6 metabolism pathway and were previously reported to be reduced in OVX results. Over recent years, an increasing body of research studies have demonstrated that a low dietary intake of vitamin B6, or low levels of vitamin B6 in the blood, might represent a novel and potentially modifiable risk factor for osteoporosis (Wang et al., 2019). Phenylalanine and hippuric acid are known to be the key biomarkers associated with the metabolic pathway of phenylalanine. Phenylalanine is an essential amino acid in the human body and belongs to the family of aromatic amino acids (Lopansri et al., 2006). Phenylalanine is oxidized by phenylalanine hydroxylase to form tyrosine and a range of brain chemicals, including levodopa, epinephrine, norepinephrine, and thyroid hormones (Fernstrom and Fernstrom, 2007). Therefore, the metabolic status of phenylalanine is closely related to normal physiological function in the body. Previous researchers (Tao et al., 2017) have highlighted that the regulation of metabolic disorders associated with phenylalanine can prevent the occurrence of osteoporosis. Hippuric acid, a glycine conjugate of benzoic acid, is expressed in many disorders associated with enzymatic metabolism, including diabetes, obesity, and osteoporosis. Hippuric acid is mainly excreted *via* glomerular filtration and by an active secretion mechanism in the renal tubules. However, when the kidney is damaged, the content of hippuric acid in the urine decreases; consequently, there is an increase in the serum levels of hippuric acid (Wang and Bao, 2013). Vitamin D needs to be catalyzed by 1α-hydroxylase in the kidney and is then hydroxylated again to become the more active 1,25-(OH)2D3. A reduction in 1,25-(OH)2D3 content will inevitably lead to the loss of bone mass and induce osteoporosis in the bones (Lin et al., 2008).

CYP19A1, an aromatase-encoding gene, is known to be related to osteoporosis and can influence BMD by altering the levels of estrogen (Sowers et al., 2006). HSD11B1 is known to be expressed by human osteoblasts *in vivo* and could reduce the proliferation of osteoblasts and induce the differentiation of osteoblasts *in vitro*. Gene polymorphism can result in direct changes in the mass of bone and can help to convert androgens and glucocorticoids to estrogen (Hwang et al., 2009). A previous study showed significant associations between a range of HSD11B1 alleles and haplotypes and bone fracture density and BMD in postmenopausal women. VDR is the target receptor that regulates the transcription of vitamin D. Previously, researchers have focused on the relationship between VDR and PMOP and demonstrated that changes in the VDR genotype were evident in patients with PMOP (Zhang L. et al., 2018). We found that the levels of these indices in the OVX group were much higher than those in the sham group, but were down regulated in the drug-treatment groups, thus suggesting that TBD can treat PMOP by regulating the specific levels of these targets.

Previous literature has reported a clear relationship between vitamin B6 levels, vitamin D, and parathyroid hormone (PTH) (Khundmiri et al., 2016), and a positive correlation between VDR, PTH, and vitamin D. This view makes the results of network pharmacology and metabolomics organically combined. Phyridoxmine and pyridoxal are two forms of vitamin B6, which are important different metabolites in the vitamin B6 metabolic pathway (Saito, 2006). In other words, there is a close connection between the gene VDR and the differential metabolites compound phyridoxmine, pyridoxal, and the vitamin B6 metabolic pathway. We used network pharmacology and metabolomics to integrate the crucial targets, differential metabolite and metabolic pathways to explore the mechanism of TBD to treat PMOP. Our analysis showed that VDR and vitamin B6 metabolism were critical mechanisms underlying the effect of TBD on PMOP. However, we have not been able to identify the specific components of TBD that combine with the target VDR. In our future research, we aim to use online docking software in an attempt to identify the specific compounds in TBD that bind to VDR; such work should provide us with stronger evidence for the wider application of TBD in the clinical treatment of PMOP.

## Conclusion

By integrating the results derived from network pharmacology and metabolomics, we identified VDR and vitamin B6 metabolism as the key indicator and pathway, respectively, for the action of TBD against PMOP. ELISAs further showed that several crucial targets (VDR, HSD11B1, and CYP19A1) were elevated in the OVX group but were reduced following TBD treatment. From a mechanistic point of view, TBD appears to regulate vitamin B6 metabolism by altering VDR content, thus mitigating the process of PMOP. Our findings enhance our understanding of how TBD could be used to treat PMOP and will help to facilitate the wider clinical use of TBD treatments.

## Data Availability Statement

The raw data supporting the conclusions of this article will be made available by the authors, without undue reservation, to any qualified researcher.

## Ethics Statement

The animal study was reviewed and approved by Animal Ethics Committee of Inner Mongolia Medical University (Reference: SCXK2015-0001).

## Author Contributions

PX conceived of and designed the experiments. FY performed the experiment and carried out the animal experiments. XD performed the data analysis and wrote the paper. FX gave some advices. JL, FM, JKL, CL, and RB collected *Echinops latifolius* Tausch samples outside. FY and XD conducted the data interpretation. All authors contributed to the article and approved the submitted version.

## Funding

This work was financially supported by National Natural Science Foundation of China (81860756), National Natural Science Foundation of China (81960758), Natural Science Foundation of Inner Mongolia Autonomous Region (2017MS08122), Natural Science Foundation of Inner Mongolia Autonomous Region (2019MS08111), Inner Mongolia Science and Technology Innovation Guide Project (02039001), Inner Mongolia Autonomous Region Higher Education Science Research Project (NJZY19099).

## Conflict of Interest

The authors declare that the research was conducted in the absence of any commercial or financial relationships that could be construed as a potential conflict of interest.
