# Supplementary material for: The Interventional Effects of Tubson-2 Decoction on Ovariectomized Rats as Determined by a Combination of Network Pharmacology and Metabolomics
Source: Front Pharmacol. 2020 Oct 14;11:581991. doi: 10.3389/fphar.2020.581991 (PMC7593846; doi:10.3389/fphar.2020.581991)
Supplement: Supplementary file 1 [file DataSheet_1.docx]

Supplementary Material

# 1.1 Supplementary Figure


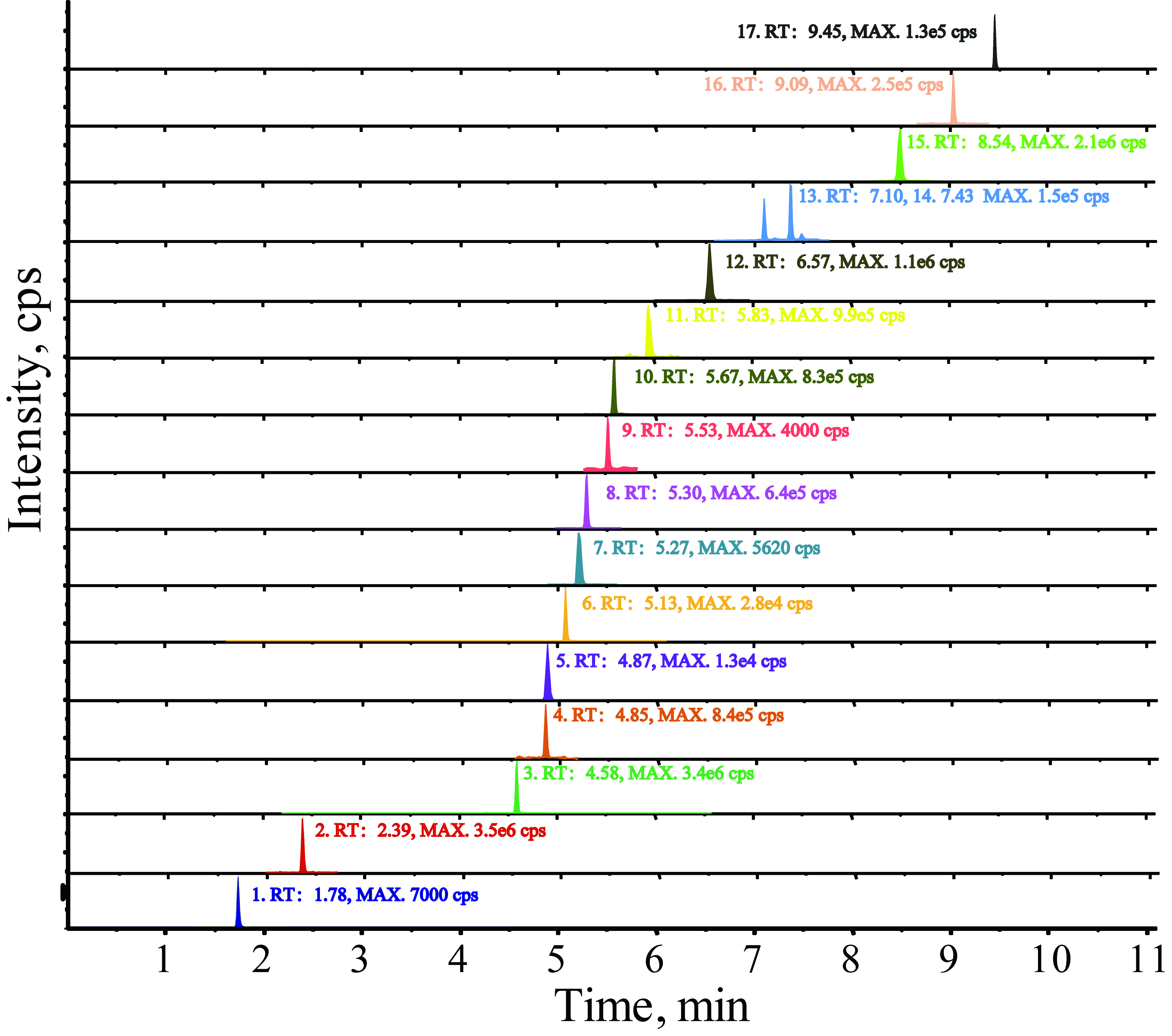


**Supplementary Figure S1.** The chemical proﬁle of TBD was achieved by UPLC-MS/MS.

## 1.2 Supplementary Table

Table S1. The content of sixteen active ingredients in Tabson-2 decoction sample

| Compound | Content (mg/g) |
| --- | --- |
| isochlorogenic acid B | 0.1295 |
| isochlorogenic acid A | 0.1843 |
| protocatechuic acid | 0.0103 |
| gentianic acid | 0.0264 |
| caffeic acid | 0.0461 |
| fumalic acid | 0.0155 |
| L-epicatechin | 0.0099 |
| cryptochlorogenic acid | 0.1355 |
| neochlorogenic acid | 0.1249 |
| chlorogenic acid | 0.1372 |
| geniposidic acid | 0.4063 |
| baicalin | 0.1277 |
| pinoresinol diglucoside | 0.7886 |
| astragalin | 0.0007 |
| aucubin | 3.3596 |
| deacetylasperulosidic acid | 0.2132 |

Table S2. The The characteristic components in TBD were analyzed with UHPLC-MS

| NO. | Compounds | Molecular formula | [M-H]-/ [M+H]+ | Retention time(min) | | |
| --- | --- | --- | --- | --- | --- | --- |
|  |  |  |  | TBD | EC | ELT |
| 1 | PYG | C6H6O3 | 125.0244 | 35.55 | 35.55 | 35.58 |
| 2 | Mairin | C30H48O3 | 455.3530 | 5.95 | 5.94 |  |
| 3 | Kaempferol | C15H10O6 | 285.0404 | 90.14 |  | 90.08 |
| 4 | Syringetin | C17H14O8 | 345.0615 |  |  | 106.58 |
| 5 | Skimmetin | C9H6O3 | 161.0244 |  | 35.59 | 35.56 |
| 6 | Protocatechuic acid | C7H6O4 | 153.0193 | 26.89 | 26.90 |  |
| 7 | EIC | C20H42 | 281.3213 | 11.38 |  |  |
| 8 | LOLIOLIDE | C11H16O3 | 195.1026 | 55.75 | 55.73 | 55.76 |
| 9 | MAE | C12H12N2O2S | 247.0546 |  |  | 71.57 7.56 |
| 10 | Heriguard | C16H18O9 | 353.0878 | 35.55 | 35.57 | 35.58 |
| 11 | Ferulaldehyde | C10H10O3 | 193.0506 | 36.78 | 36.81 |  |
| 12 | Coniferol | C10H12O3 | 179.0713 | 5.14  39.89 | 5.15 39.91 |  |
| 13 | HYKOP | C9H10O4 | 181.0506 | 38.24 | 38.26 |  |
| 14 | Rutin | C27H30O16 | 609.1461 | 59.07 |  | 59.13 |
| 15 | Isomangiferin | C19H18O11 | 421.0776 | 35.55 | 35.57 | 35.6 |
| 16 | Genistein | C15H10O5 | 269.0455 | 77.58 85.24 |  | 77.70 85.30 |
| 17 | Ursolic acid | C9H10O2 | 149.0608 | 24.39 | 24.47 | 24.38 |
| 18 | Astragalin | C21H20O11 | 447.0932 | 64.87 |  | 65.98 |
| 19 | Ombuin | C17H14O7 | 329.0666 | 8.06 | 8.03 |  |
| 20 | Ent-Epicatechin | C15H14O6 | 289.0717 | 26.54 | 26.22 |  |
| 21 | Yangambin | C24H30O8 | 445.1867 | 115.65 |  | 113.48 |
| 22 | (+)-Tabernemontanine | C21H26N2O3 | 353.1870 | 35.55 | 35.57 | 35.58 |
| 23 | MHP | C16H23NO4 | 292.1554 | 14.02 |  | 14.02 |
| 24 | (2R,3S)-2-amino-3-hydroxy-succinic acid | C4H7NO5 | 148.0251 | 10.64 |  |  |
| 25 | ZINC00394284 | C10H14O4 | 197.0819 | 34.48 | 34.48 |  |
| 26 | Helenalin | C15H18O4 | 261.1132 | 108.54 |  | 108.43 |
| 27 | FER | C10H10O4 | 193.0506 | 53.28 | 53.25 | 53.37 |
| 28 | HMF | C6H6O3 | 125.0244 | 35.55 | 35.55 | 35.58 |
| 29 | LINALOOL (D) | C10H18O | 153.1284 | 26.89 | 26.90 | 26.90 |
| 30 | Octanol | C8H18O | 129.1284 | 12.18 | 12.78 | 12.78 |
| 31 | 2,6-Dimethoxyquinol | C8H10O4 | 169.0506 | 19.16 20.27 | 19.16 20.26 |  |
| 32 | M-COUMARIC ACID | C9H8O3 | 163.0400 | 50.61 | 50.65 | 50.61 |
| 33 | 11-Deoxoglycyrrhetinic acid | C30H48O3 | 455.3530 | 5.95  7.58 | 5.94  7.58 |  |
| 34 | 5-Hydroxymethyl Furaldehyde | C12H9BrO3 | 278.9662 | 12.6 | 12.56 |  |
| 35 | Syringin | C17H24O9 | 371.1347 | 5.95 | 5.94 | 5.40 |
| 36 | Koaburaside | C14H2OO9 | 328.9575 | 18.79 |  | 18.82 |
| 37 | Berberine | C20H18NO4 | 335.1163 | 29.68 | 29.28 |  |
| 38 | Isoquercitrin | C21H20O12 | 463.0881 | 55.84 |  | 55.95 |
| 39 | Cyrtomin | C17H16O6 | 315.0874 | 26.67 | 26.67 | 26.66 |
| 40 | Geniposide | C17H24O10 | 387.1296 | 30.86 | 30.87 | 30.9 |
| 41 | Geniposidic acid | C16H22O10 | 373.1140 | 25.74 | 25.74 |  |
| 42 | PDG | C32H42O16 | 681.2396 | 41.38 | 41.34 |  |
| 43 | Aucuboside | C15H22O9 | 345.1191 | 29.25 |  | 29.24 |
| 44 | Apigenin7-O-glucoside | C21H20O10 | 431.0983 | 70.46 | 70.45 |  |
| 45 | Apigenin-7-O-β-D-(-6″-p-coumaroyl)-glucoside | C30H26O12 | 577.1351 | 94.55 | 94.54 |  |
| 46 | 3,4-dicaffeoyl quinic acid | C25H24O12 | 515.1194 | 69.56 |  | 69.56 |
| 47 | ethylcaffeate | C11H12O4 | 207.0662 | 45.31 |  | 45.24 |
| 48 | 1,5-dicaffeoyl quinic acid | C25H24O12 | 515.1195 | 69.56 | 69.65 | 69.56 |
| 49 | 3,5-dicaffeoyl quinic acid | C25H24O12 | 515.1195 | 60.26 | 60.27 | 60.27 |
| 50 | Methyl3,4-dicaffeoylquinicacid | C26H27O13 | 546.1378 | 45.2 |  | 45.2 |
| 51 | Methyl3,5-dicaffeoylquinicacid | C26H27O13 | 546.1378 | 45.2 |  | 45.2 |
| 52 | Methyl-1-O-methyl3,5-dicaffeoylquinicacid | C27H28O12 | 543.1507 | 20.12 | 20.10 |  |
| 53 | 3,4-Dihydroxybenzoic acid | C11H12O4 | 207.0662 | 48.19 | 48.18 |  |
| 54 | Isochlorogenic acid A | C25H24O12 | 515.1195 | 61.66 | 61.27 |  |
| 55 | Isochlorogenic acid C | C25H24O12 | 515.1195 | 61.66 |  | 61.27 |
| 56 | Neochlorogenic acid | C16H18O9 | 353.0878 | 35.55 | 35.57 | 35.58 |
| 57 | [Cryptochlorogenic acid](https://www.chem960.com/cassearch/casalias/40207?AliasCatalog=%E8%8B%B1%E6%96%87%E5%88%AB%E5%90%8D) | C16H18O9 | 353.0878 | 35.55 28.16 | 35.57 28.19 37.13 | 35.58 28.20 |
| 58 | [Quercetin-3-rhamnoside](https://www.chem960.com/cassearch/casalias/37989?AliasCatalog=%E8%8B%B1%E6%96%87%E5%88%AB%E5%90%8D) | C21H20O11 | 447.0932 | 55.87 |  | 55.93 |
| 59 | Quercetin | C15H10O7 | 301.0353 | 7.80 9.33 |  | 7.80 9.35 |
| 60 | Caffeic acid | C9H8O4 | 179.0350 | 39.88 | 39.91 | 39.9 |
| 61 | Chlorogenic acid | C16H18O9 | 353.0878 | 35.55 28.16 | 35.57 28.19 | 35.58 28.20 |
| 62 | β-Sitosterol | C29H50O | 413.3788 | 44.17 | 44.11 | 44.15 |
