# Supplementary material for: The Interventional Effects of Tubson-2 Decoction on Ovariectomized Rats as Determined by a Combination of Network Pharmacology and Metabolomics
Source: Front Pharmacol. 2020 Oct 14;11:581991. doi: 10.3389/fphar.2020.581991 (PMC7593846; doi:10.3389/fphar.2020.581991)
Supplement: Supplementary file 2 [file DataSheet_2.docx]

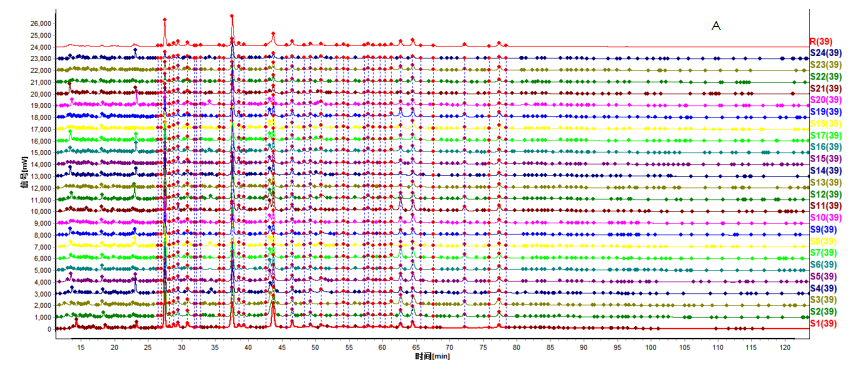


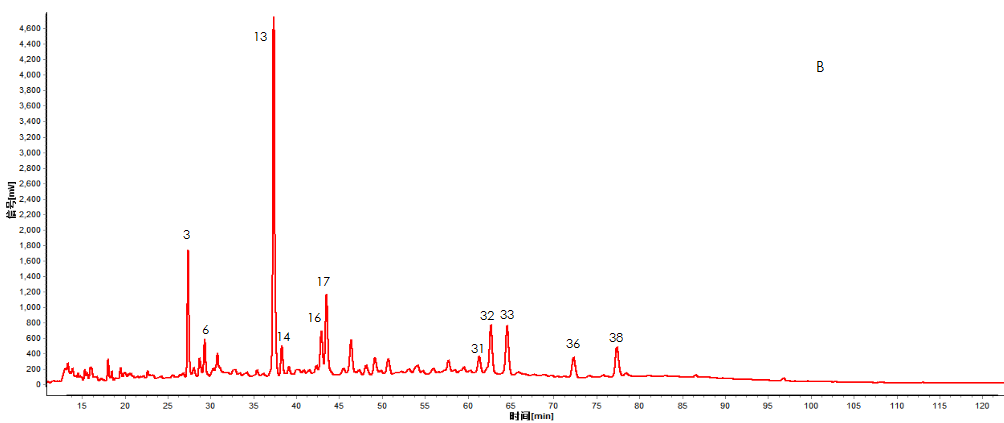

C

38

36

33

32

17

16

13

6

3

Figure 1: A:Fingerprints of the Tabson-2 decoction raw extract in 24 batches, B:Common peak of characteristic maps，C:HPLC chromatogram of mixed reference standards （3: geniposide acid; 6: neochlorogenic acid; 13: chlorogenic acid; 16: geniposide; 17: pinoresinol diglucoside; 32: isochlorogenic acid A; 33: 1,5-O -Biscaffeoylquinic acid; 36: isochlorogenic acid C; 38: Astragalin）The peak numbers are in accordance with the compound numbers in Table 1.

Table 1 Contents of nine analytes in 24 batches of Tabson-2 decoction raw extract samples (mg/g).

| Batch number | 3 | 6 | 13 | 16 | 17 | 32 | 33 | 36 | 38 |
| --- | --- | --- | --- | --- | --- | --- | --- | --- | --- |
| 1 | 0.100 | 0.086 | 0.587 | 0.021 | 0.140 | 0.116 | 0.070 | 0.042 | 0.025 |
| 2 | 0.089 | 0.090 | 0.596 | 0.032 | 0.152 | 0.188 | 0.085 | 0.105 | 0.014 |
| 3 | 0.108 | 0.096 | 0.281 | 0.088 | 0.191 | 0.263 | 0.066 | 0.120 | 0.011 |
| 4 | 0.150 | 0.084 | 0.406 | 0.093 | 0.146 | 0.152 | 0.080 | 0.068 | 0.021 |
| 5 | 0.060 | 0.109 | 0.240 | 0.109 | 0.177 | 0.207 | 0.060 | 0.088 | 0.026 |
| 6 | 0.129 | 0.083 | 0.423 | 0.085 | 0.129 | 0.012 | 0.062 | 0.050 | 0.011 |
| 7 | 0.094 | 0.085 | 0.185 | 0.087 | 0.139 | 0.268 | 0.063 | 0.113 | 0.012 |
| 8 | 0.080 | 0.106 | 0.488 | 0.087 | 0.142 | 0.142 | 0.051 | 0.067 | 0.010 |
| 9 | 0.088 | 0.092 | 0.343 | 0.071 | 0.124 | 0.125 | 0.049 | 0.044 | 0.016 |
| 10 | 0.087 | 0.093 | 0.380 | 0.074 | 0.127 | 0.134 | 0.050 | 0.046 | 0.025 |
| 11 | 0.057 | 0.109 | 0.241 | 0.107 | 0.187 | 0.322 | 0.035 | 0.160 | 0.020 |
| 12 | 0.075 | 0.107 | 0.176 | 0.098 | 0.168 | 0.222 | 0.056 | 0.110 | 0.033 |
| 13 | 0.090 | 0.100 | 0.174 | 0.078 | 0.145 | 0.151 | 0.083 | 0.053 | 0.024 |
| 14 | 0.071 | 0.092 | 0.422 | 0.073 | 0.144 | 0.098 | 0.047 | 0.030 | 0.016 |
| 15 | 0.060 | 0.099 | 0.192 | 0.073 | 0.139 | 0.154 | 0.068 | 0.086 | 0.020 |
| 16 | 0.103 | 0.096 | 0.467 | 0.078 | 0.147 | 0.234 | 0.063 | 0.130 | 0.021 |
| 17 | 0.106 | 0.097 | 0.359 | 0.072 | 0.125 | 0.157 | 0.062 | 0.054 | 0.021 |
| 18 | 0.083 | 0.103 | 0.425 | 0.080 | 0.132 | 0.107 | 0.050 | 0.051 | 0.030 |
| 19 | 0.055 | 0.103 | 0.784 | 0.076 | 0.133 | 0.230 | 0.056 | 0.096 | 0.016 |
| 20 | 0.048 | 0.091 | 0.349 | 0.055 | 0.087 | 0.089 | 0.046 | 0.048 | 0.008 |
| 21 | 0.059 | 0.103 | 0.334 | 0.035 | 0.090 | 0.050 | 0.032 | 0.015 | 0.013 |
| 22 | 0.040 | 0.079 | 0.280 | 0.032 | 0.074 | 0.062 | 0.053 | 0.022 | 0.021 |
| 23 | 0.091 | 0.071 | 0.290 | 0.022 | 0.067 | 0.084 | 0.047 | 0.035 | 0.020 |
| 24 | 0.083 | 0.066 | 0.277 | 0.022 | 0.058 | 0.050 | 0.031 | 0.018 | 0.009 |
